# Sensitivity and specificity of a rapid, point-of-care assay distinguishing bacterial from viral infections in acute care settings

**DOI:** 10.1128/jcm.00262-26

**Published:** 2026-10-27

**Authors:** Maria E. Sundaram, Taylor A. Wahlig, Sophie Arbefeville

**Affiliations:** 1 Center for Clinical Epidemiology and Population Health, Marshfield Clinic Research Institute 513992, Marshfield, Wisconsin, USA; 2 Microbiology & Molecular Pathology, Marshfield Labs, Sanford Health Marshfield Clinic, Marshfield, Wisconsin, USA; Endeavor Health, Evanston, Illinois, Chicago, USA

**Keywords:** bacterial infection, viral infection, MeMed BV assay, bacteremia

## Abstract

Differentiating between bacterial and viral infections is essential in the emergency department (ED) to guide appropriate antibiotic treatment. Recent assays, such as the Food and Drug Administration (FDA)-cleared MeMed BV assay, can help with this distinction. The MeMed BV assay measures three host immune proteins—Tumor necrosis factor (TNF)-related apoptosis-induced ligand, Interferon (IFN)-γ-induced protein 10, and C-reactive protein—in blood samples. The results are combined into a score from 0 to 100, where a higher score indicates a higher likelihood of a bacterial infection and a lower score suggests a viral infection or other non-bacterial causes. The study evaluates the feasibility of using the MeMed BV assay-generated score to help clinicians decide whether to order blood cultures in emergency departments, as well as assessing the accuracy of the MeMed BV assay in distinguishing between bacterial and viral infections in ED patients. The assay’s performance was also compared with standard biomarker tests and white blood cell (WBC) counts for predicting bacterial infections. This study included 203 illness episodes, of which a total of 108 were associated with bacterial infection (25 had a positive blood culture; 107 had a non-blood sample positive for a bacterial pathogen) and 18 were associated with a viral pathogen. Combined, the MeMed BV assay’s “high bacterial likelihood” and “moderate bacterial likelihood” scores yielded a sensitivity and specificity of 87% and 32%, respectively, in correctly identifying any bacterial infection; the scores had a sensitivity and specificity of 95% and 25%, respectively, in correctly identifying bacteremia. The future of differentiating between bacterial and viral infections is moving toward integrated diagnostic methods. To address antibiotic resistance, future diagnostics will leverage host immune response biomarkers, advanced molecular panels, and machine learning techniques. This will enable rapid and accurate results at the point of care.

**IMPORTANCE:**

Distinguishing between bacterial and viral infections in healthcare settings can be challenging, as patients often present with similar symptoms and clinical features. In recent years, several new biomarkers have emerged as promising targets for algorithm-based tests designed to differentiate between these types of infections. An increasing number of FDA-approved assays that utilize these biomarkers have entered the market, employing various algorithms. These tests are being used in different clinical settings, such as emergency rooms, to assist clinicians in making accurate diagnoses and prescribing antibiotics appropriately. It is essential for clinicians to correctly order and interpret the results of these assays to fully realize their potential. Our study demonstrates that when such an assay is not used within the FDA-approved context, it does not achieve its full effectiveness.

## INTRODUCTION

Distinguishing between infections with a bacterial vs viral etiology in healthcare settings can be challenging, as patients often present with similar symptoms and disease presentation. In many cases, the specific etiology cannot be appropriately ascertained without pathogen-specific testing, including nucleic acid amplification tests (NAATs) and/or culture. Although pathogen-specific tests provide helpful insight into treatment plans, prevent the overuse of antibiotics for non-bacterial infections, and ensure that patients receive appropriate treatment, they may be both time-consuming and expensive.

The distinction between infections with bacterial vs viral etiology is especially critical in the emergency department (ED) and other environments where rapid response is beneficial, as it helps in the appropriate and timely administration of antibiotics. In EDs, blood samples for culture may be collected from patients suspected of having infections, even if their clinical presentation has a low likelihood of being associated with bacteremia. However, contamination rates for blood cultures collected in the ED are higher compared to inpatient settings (1, 2). Additionally, managing false-positive blood culture results can lead to unnecessary prolonged antibiotic treatment, which increases the risk of bacterial resistance, incurs higher pharmacy and laboratory costs, causes processing delays, and results in longer hospital stays.

In some cases, physiological changes in a patient’s response to infection—such as alterations in body temperature, heart rate, and specific relevant biomarkers such as C-reactive protein (CRP) and procalcitonin—can help determine bacterial vs viral etiology before blood culture results are provided (3–5). Several biomarkers have been assessed for potential utility in distinguishing between bacterial vs viral etiology. However, no single biomarker appears to be consistently accurate in the differentiation of bacterial vs viral etiology (6). It is possible that combining multiple biomarkers in an integrated algorithm may improve predictive capacity to distinguish between bacterial and viral infections.

Several algorithm-based assays have been developed for use in EDs to help clinicians prescribe antibiotics appropriately. One such assay is the MeMed BV test (7) (MeMed BV, DiaSorin, USA). MeMed BV, which is available as a rapid, point-of-care test, measures host immune proteins that may be indicative of bacteremia (bacterial infection in blood samples). These proteins are TNF-related apoptosis-induced ligand (TRAIL), IFN-γ-induced protein 10 (IP-10), and C-reactive protein. Using immunoassay methodology, the results are combined into a single score (0 to 100) that is then categorized into five groups, which are on a scale from high likelihood of bacterial infection to high likelihood of viral infection.

We investigated the use of this assay in an acute care setting in Marshfield, Wisconsin. The primary objective of this study was to identify the feasibility of using the MeMed BV assay-generated score to assist clinicians in deciding whether to order blood cultures in acute care settings. We additionally assessed the accuracy of the MeMed BV assay in categorizing patients as having either bacterial or viral infection. Finally, we compared the results of standard biomarker tests and white blood cell (WBC) counts with MeMed BV score results to assess the predictive capacity of the MeMed BV assay compared to biomarkers alone.

## MATERIALS AND METHODS

### Setting and participants

Marshfield Clinic Health System (MCHS) is a large, rural health system serving central and northern Wisconsin and the Upper Peninsula of Michigan, with a large-scale clinical laboratory system (Marshfield Labs) offering a broad range of clinical testing, including a full clinical microbiology test menu in bacteriology, mycobacteriology, mycology, and molecular infectious diseases.

Individuals were included in this analysis if they were patients in a Marshfield Clinic Health System emergency department receiving clinical care between 1 December 2023 and 31 July 2024, with a blood culture collected as part of their clinical management.

### Samples collected for clinical testing

For inclusion in the analysis, blood cultures had to have been obtained via a properly documented peripheral venipuncture. Two sets of blood bottles were required for each peripheral venipuncture draw (one aerobic and one anaerobic bottle per set, for a total of four bottles). Each bottle was required to contain 8 to 10 mL of blood, resulting in a total of 32 to 40 mL from both sets. Blood cultures with smaller volumes were not eligible for inclusion in this analysis.

If individuals contributed more than one blood culture order within the same 2-week period, these were assumed to represent the same illness episode, and only the first blood culture was used for our analysis.

For some patients, additional specimen types, such as respiratory, fecal, urine, wound tissue, or specimen swabs when appropriate, were collected during routine clinical care for testing for infectious agents. Where available, we also included the results from these clinical tests in our analysis as evidence of a bacterial infection.

In addition to having a blood culture ordered in the ED, a minimum of 1 mL of serum had to remain from a serum specimen collected at the same time as part of the clinical care and had not been refrigerated at 2–8°C for longer than 8 h after collection.

### Classification of “bacteremia,” “true bacterial,” and “true viral” infections

#### Bacteremia

Blood culture results were used to determine whether patients had true bacteremia. This was accomplished using the BACT/ALERT VIRTUO, a fully automated blood culture system. The BIOFIRE Blood Culture Identification 2 (BCID2) panel (bioMérieux) (8) was used to rapidly identify 43 targets, including gram-positive/negative bacteria, yeast, and 10 antimicrobial resistance genes directly from positive blood cultures. Additionally, the VITEK MS (bioMérieux) was used to confirm the identifications made by the BCID2 panel and to identify organisms not detected by the BCID2 (9). A blood culture was classified as true bacteremia when more than one blood bottle tested positive for the same pathogen or when a clinically significant pathogen was identified from a single bottle. In contrast, a blood culture was considered a false positive (i.e., not true bacteremia) if only one bottle showed a positive result for an organism that was part of the normal human skin flora, and the other bottle showed a negative result. All patients with bacteremia were also considered to have true bacterial infection.

#### Bacterial infection

Clinical test results from specimen types other than blood cultures were also used to determine whether patients had a true bacterial vs viral infection (not necessarily bacteremia). Bacterial pathogens from non-blood samples were identified using a combination of traditional aerobic/anaerobic/fungal culture and identified with the VITEK MS or detected with nucleic acid amplification tests ordered as part of clinical care (10). Patients were considered to have a true bacterial infection if a non-blood sample was found to contain pathogenic bacteria; however, patients were only considered to have bacteremia if pathogenic bacteria were identified in blood culture. We considered non-specific clinical results, such as “microbial growth,” “mixed microbiota,” and “mixed skin flora,” to represent either normal skin flora or sample contamination rather than pathogenic involvement.

#### Viral infection

Viral pathogens were identified based on results from PCR or other nucleic acid amplification tests ordered as part of clinical care. Influenza A/B, Respiratory syncytial virus (RSV), and Severe acute respiratory syndrome coronavirus 2 (SARS-CoV-2) were identified in the clinical setting using a combination of the BIOFIRE Respiratory 2.1 Panel (bioMérieux) (11), the Xpert Xpress CoV-2/Flu/RSV plus (Cepheid) (12), and the Panther Fusion SARS-CoV-2/Flu A/B/RSV assay (Hologic) (13). Additional respiratory pathogens (adenovirus, seasonal coronavirus, enterovirus/rhinovirus, human metapneumovirus, and parainfluenza virus 1–4) were identified using the BIOFIRE Respiratory 2.1 Panel; gastrointestinal pathogens (adenovirus, astrovirus, norovirus, rotavirus, and sapovirus) were identified using the BIOFIRE Gastrointestinal Panel (bioMérieux) (14) and BD MAX Enteric Viral Panel (Becton Dickinson) (15). Additional parasites (*Cryptosporidium*, *Cyclospora*, *Entamoeba*, and *Giardia*) were identified using a combination of the BIOFIRE Gastrointestinal Panel and the BD MAX Enteric Parasite Panel (Becton Dickinson) (16).

### MeMed BV categorization

After clinical testing, serum specimens were frozen at −80°C for no longer than 12 weeks, in accordance with the assay package insert requirements, before thawing and testing to obtain the MeMed BV score, using the DiaSorin XL-LIAISON instrument.

Categorized results provided by the assay correspond to categorized values of scores: HighVIR (high likelihood of viral infection or other non-bacterial etiology, 0–10); ModVIR (moderate likelihood of viral infection or other non-bacterial etiology, 11–34); EQV (equivocal, 35–65); ModBACT (moderate likelihood of bacterial infection, 66–89); and HighBACT (high likelihood of bacterial infection, 90–100).

The performance of the FDA-approved MeMed assay was verified in the testing laboratory prior to evaluating the study samples. Additionally, an applications specialist from DiaSorin provided training to the personnel conducting the MeMed BV testing.

### Additional patient information

In addition to serum samples and results from clinical testing, we also collected the following information regarding illness episodes from the electronic health record for analysis: length of stay (in days); chest X-ray or CT performed (yes/no); vital signs (temperature in degrees Fahrenheit, oxygen saturation, pulse and respiratory rates, and blood pressure); and relevant biomarkers collected as part of clinical care (procalcitonin, C-reactive protein, and white blood cell counts). We additionally collected demographic characteristics (sex, age in years at the time of presentation, race and ethnicity, and body mass index [BMI]).

### Statistical analysis

The primary statistical analysis compared MeMed BV assay results to a gold standard of true bacterial infection, defined as the presence of bacterial pathogens in blood or in non-blood samples of patients either recovered in culture or identified by NAAT by the clinical laboratory at Marshfield Clinic. We calculated the specificity and sensitivity of the MeMed BV assay for the presence of bacterial and viral pathogens based on these two gold standards and calculated Wald 95% confidence intervals. Receiver operating characteristic (ROC) curves and areas under the curve (AUCs) were then calculated separately for bacterial and viral outcomes.

Secondarily, we used these gold standards to estimate a second set of ROC curves using maximum-likelihood probit models, which identified estimated differences in AUC values according to patient age at the time of testing (measured at cut-points of 20, 40, 60, and 80 years) and sex (male vs female). Maximum-likelihood probit models were univariate models with only the MeMed score as a predictor of the gold-standard outcome.

Finally, we compared the predictive capacity of different laboratory-based predictors for bacterial vs non-bacterial (i.e., viral result or pan-negative result), using generalized linear models with a log link and Poisson distribution with robust variance estimation. The first of these models (“Model A”) used log-transformed procalcitonin, white blood cell count, and CRP as predictors; the second (“Model B”) used the 0–100 score yielded by the MeMed assay with no other covariates; and the third (“Model C”) used the categorized result provided by the MeMed assay with no other covariates. The predictive capacity of these models was compared using Akaike information criterion (AIC) and Bayesian information criterion (BIC) values. All statistical analyses were completed using Stata version 19 (Cary, NC).

In a sensitivity analysis, we assessed the performance of the MeMed BV assay against a refined gold standard of true bacteremia. In this analysis, the gold standard outcome was restricted to individuals with bacteria-positive blood cultures only; individuals with negative blood cultures but positive non-blood samples were excluded from this analysis.

## RESULTS

From 1 December 2023 to 31 July 2024, there were 203 illness episodes that met the study’s inclusion criteria; there were six individuals who contributed multiple samples during separate illness episodes. The median Interquartile range (IQR) age of study participants was 69 (57, 78) years; 40% were female, and 79% had a BMI ≥ 35 (Table 1).

**TABLE 1:** Demographic characteristics of the study population

Characteristic	True bacterial infection (*N* = 108)	True viral infection (*N* = 18)	No confirmed bacterial or viral infection (*N* = 82)	Total study population (*N* = 203)^*a*^
Age, median (IQR)	70 (55, 79)	65 (57, 77)	69 (59, 78)	69 (57, 78)
Sex: *N* (%)				
Female	47 (43.5)	8 (44.4)	30 (36.6)	82 (40.4)
Male	61 (56.5)	10 (55.6)	52 (63.4)	121 (59.6)
BMI ≥ 35: *N* (%)	82 (75.9)	16 (88.9)	67 (81.7)	160 (78.8)

^*a*^ Total study population is less than the sum of bacterial infections plus viral infections plus pan-negative individuals because five individuals had coinfections with a bacterial and a viral pathogen.

**Fig 1:**
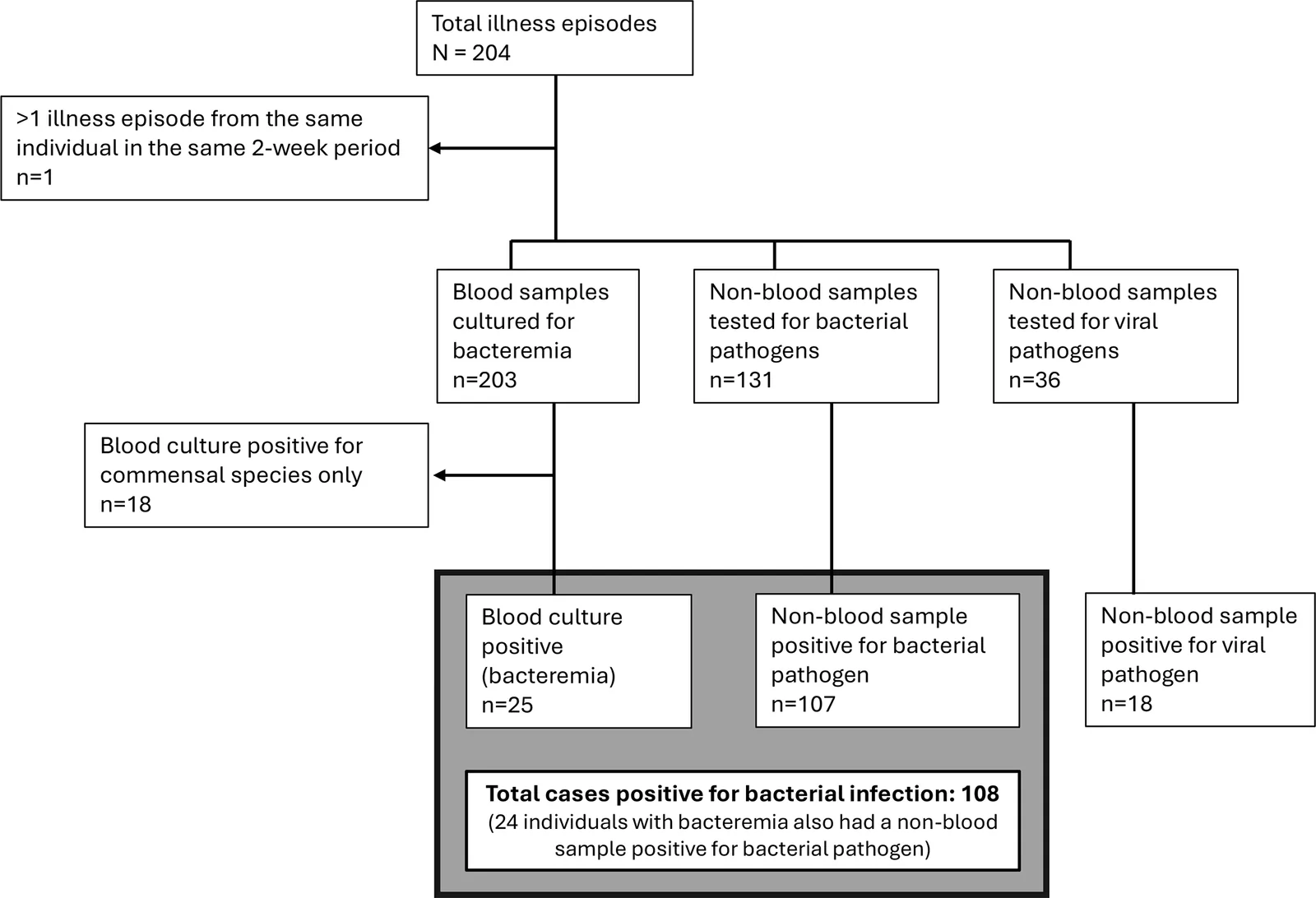
Flow diagram of illness episodes, tests performed, and test results.

All 203 episodes of illness (“cases”) included in the analysis were assessed for bacteremia; an additional 131 were tested for the presence of a bacterial infection in a non-blood sample, 36 were tested for the presence of a viral infection in a non-blood sample, and 2 were tested for the presence of a parasitic infection. Of the 203 cases, a total of 108 were associated with true bacterial infection; 18 were associated with true viral infection based on clinical PCR-based testing; and 82 cases were not associated with any pathogenic bacterial or viral infection. There were five cases associated with bacterial-viral coinfections. There were also nine cases in the study associated with a fungal pathogen identified. Of these, all nine also had a bacterial pathogen; one had a bacterial-viral coinfection. Of the 108 cases with bacterial infection, 25 (23%; 25/108) had bacteremia (Fig. 1). There were no cases of parasitic infection.

**Fig 2:**
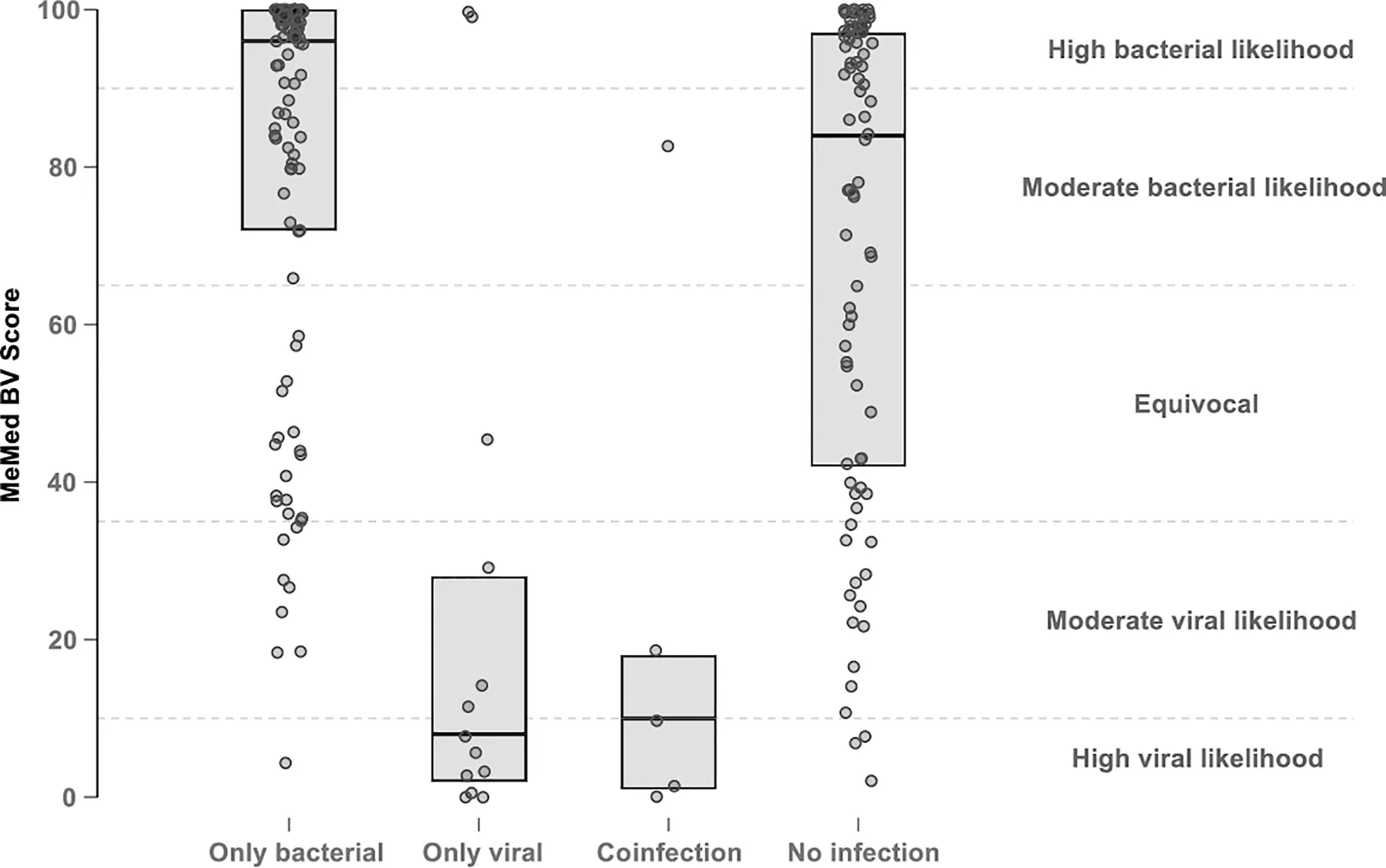
Box plot of the MeMed BV assay score assigned to individuals with only bacterial, only viral, coinfection, and no infection. Individual scores in each category are marked with dots.

**Fig 3:**
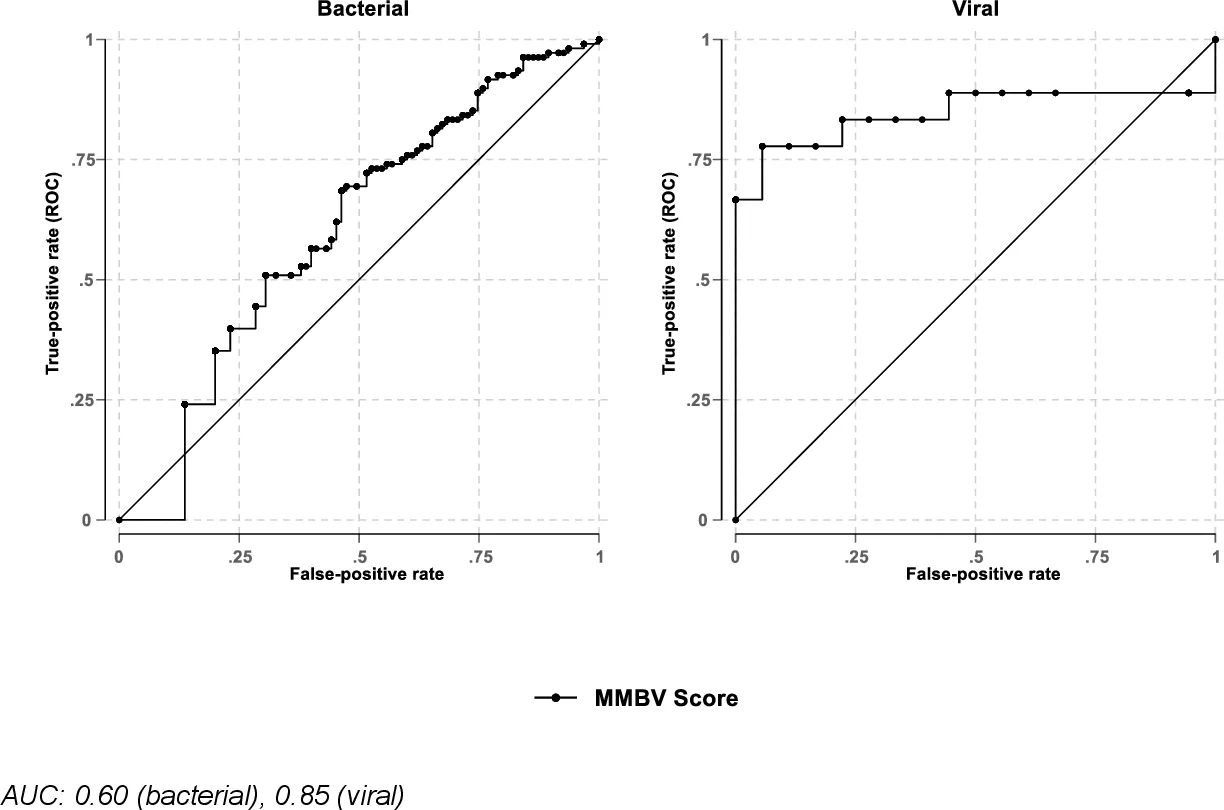
Receiver operating characteristic (ROC) curve describing the capacity of MeMed BV score in correctly predicting the presence of a bacterial (left) or viral (right) pathogen.

The assay assigned a score of 90–100 (high likelihood of bacterial infection) to 99 cases, of which 61 had confirmed bacterial infection and 19 had bacteremia (56% of total bacterial infections [61/108] and 76% of total bacteremia cases [19/25]) (Table 2; Fig. 2). The assay conversely assigned a score of 0–10 (high likelihood of viral infection) to 14 cases, of which 10 had true viral infection (56% [10/18] of total viral infections; Table 2). The assay assigned 36 cases (18%) [36/203] to an “equivocal” score. The MeMed BV assay sensitivity and specificity were calculated at 94% (93.84%; Table S1C) (95% CI: 85–98%) and 21% (20.83%; Table S1C) (95% CI: 11–35%), respectively, for identifying true bacterial infections and counting only MeMed BV results that assigned a “high” likelihood of bacterial or viral infection. Sensitivity and specificity for bacterial infections were 87% (86.81%; Table S2C) (95% CI: 78–93%) and 32% (31.57%; Table S2C) (95% CI: 21–43%), respectively, when including MeMed BV results with “moderate” likelihood of bacterial or viral infection. When restricting bacterial infections to cases of bacteremia and including MeMed BV results with “moderate” likelihood, the assay demonstrated 95% (95% CI: 77–100%) sensitivity and 25% (18–33%) specificity. These results were reflected in AUC calculations for identifying bacterial (AUC: 0.60) and viral (AUC: 0.83) infections (Fig. 3); AUC values increased with younger age and for females vs males; these differences were more substantial for bacterial outcomes (ages) or viral outcomes (sex) (Fig. S1).

**TABLE 2:** MeMed BV assay results compared to blood culture results^*c*^

	High likelihood of bacterial infection (*N* = 99)	Moderate likelihood of bacterial infection (*N* = 32)	Equivocal (*N* = 36)	Moderate likelihood of viral infection (*N* = 22)	High likelihood of viral infection (*N* = 14)	Total (*N* = 203)
Blood culture testing performed	99	32	36	22	14	203
**Blood culture positive for bacterial infection**	**19**	**2**	**3**	**1**	**0**	**25**
Blood culture likely contaminated by commensal/non-pathogenic species	10	3	2	2	1	18
**Blood culture negative for bacterial infection**	**70**	**27**	**31**	**19**	**13**	**160**
Bacterial testing performed for a non-blood sample	71	21	23	10	6	131
Sample positive for bacterial infection	60	18	17	8	4	107
**Total cases positive for bacterial infection^*a*^**	**61**	**18**	**17**	**8**	**4**	**108**
**Total cases negative for bacterial infection**	**38**	**14**	**19**	**14**	**10**	**95**
Viral testing performed for a non-blood sample	11	5	5	5	10	36
**Sample positive for viral infection^*b*^**	**2**	**1**	**1**	**4**	**10**	**18**
**Sample negative for viral infection**	**9**	**4**	**4**	**1**	**0**	**18**

^*a*^ Bacterial pathogens identified from blood culture or other testing in the clinical setting.

^*b*^ Viral pathogens identified from PCR or other testing in the clinical setting.

^*c*^ Bold values indicate the test-positive and test-negative values discussed throughout the manuscript.

Generalized linear models estimating the risk ratio of a bacterial infection identified similar predictive capacity among Model A (procalcitonin, WBC count, and C-reactive protein), Model B (the continuous MeMed BV score alone), and Model C (5-level categorized MeMed BV result). Performance metrics such as the AIC and the BIC were slightly more favorable for the MeMed model than for the model using just procalcitonin, WBC, and CRP (Table 3). However, a likelihood ratio test comparing Model A with Model A plus MeMed assay results did not indicate substantial additional benefit either for the continuous score (likelihood ratio test *χ*^2^ = 0.72; *P* = 0.40) or for the 5-level categorized assay result (likelihood ratio test *χ*^2^ = 0.95; *P* = 0.33).

**TABLE 3:** Measurements of predictive capacity of different generalized linear models with bacterial infection as the primary outcome of interest^*a*^^,*f*^

Characteristics	Risk ratio	95% CI	AIC^*b*^	BIC^*c*^
Model 1			**1.87**	**−544.57**
Procalcitonin (log-transformed)	1.03	0.94–1.12		
WBC (thousands/µL)^*d*^	1.00	0.98–1.02		
CRP (mg/L)^*e*^	1.01	0.99–1.02		
Model 2			**1.73**	**−936.21**
MeMed BV score (0 = viral, 100 = bacterial)	1.01	1.00–1.01		
Model 3			**1.74**	**−932.72**
Categorized MeMed result (1 = viral, 5 = bacterial)	1.07	0.97–1.17		

^*a*^ Models presented are generalized linear models with a log link and a Poisson distribution with robust variance estimation.

^*b*^ AIC, Akaike Information Criterion.

^*c*^ BIC, Bayesian Information Criterion.

^*d*^ WBC, white blood cell count.

^*e*^ CRP, C-reactive protein.

^*f*^ Bold values indicate the test-positive and test-negative values discussed throughout the manuscript.

An analysis investigating infectious etiology among six instances of samples associated with a personal history of immunotherapy or with individuals with a history of organ or tissue transplant indicated one instance of no bacterial growth on culture and five instances of infection with pathogenic bacteria. Among these potentially immunocompromised individuals, there was no evidence that pathogenic disease was being caused by stereotypically “commensal” bacteria.

## DISCUSSION

In an analysis comparing results from blood culture to results from MeMed BV, we identified that MeMed BV had high sensitivity but low specificity in the correct identification of bacterial pathogens (i.e., the MeMed BV assay more accurately predicted true viral infections compared to true bacterial infections). This finding suggests that the MeMed BV assay may have utility in clinical/emergent settings as a way to rule out bacterial infection by way of viral infection identification, as individuals with a high likelihood of viral infection according to the MeMed BV assay are less likely to have true bacterial infection.

A similar evaluation previously investigated the level of agreement between viral MeMed BV results and blood cultures negative for bacterial infection and found high agreement between those outcomes (17). Additionally, a study comparing the performance of the MeMed BV assay with an mRNA-based consensus transcriptome test (Triverity) to differentiate between bacterial and viral infections in adults with acute respiratory infections (18) suggested similar assay performance, with the MeMed BV assay tending to “overcall” bacterial infections.

However, another investigation of a diagnostic tool based on TRAIL, IP-10, and CRP noted higher specificity than identified in this analysis (19); additionally, two other investigations of adults with signs of lower respiratory tract infection yielded both high sensitivity and specificity for bacterial infection for the MeMed BV assay (18, 20). It is possible that these two MeMed BV studies reported higher specificity than in our study because their study designs were restricted to lower respiratory tract infection. In comparison, our study included all individuals within a specified time frame for whom a blood culture had been taken, regardless of the type of illness or suspected illness etiology.

Our results suggested that the MeMed BV score performed better in identifying true bacterial infections in the younger population. Age substantially affects the differentiation between bacterial and viral infections due to age-related changes in the immune system. In addition to functional and phenotypic alterations in immune cells, studies have shown that the circulating concentrations of inflammatory mediators in older adults are higher than those in younger individuals (21). Our findings underline that predictors that are effective for one age group may be less reliable or even misleading for another group, particularly at the extremes of age. However, individuals whose samples were tested as part of this study were largely older adults, with the number of younger adults and children substantially underrepresented.

Our comparison of MeMed BV assay results versus separate biomarkers (procalcitonin, CRP, and WBC) indicated that the MeMed BV assay accurately generated scores to distinguish between bacterial and viral infections. However, it did not appear to provide additional statistical benefit beyond models combining results from procalcitonin, CRP, and WBC count. Existing limited evidence suggests that other models, including readily available patient characteristics and routine laboratory results, may be sufficient for decision-making on safe withholding of blood culture testing in low-risk patients in the emergency room (1).

This study has several limitations. First, our study focuses on patients from whom blood samples had already been collected as part of clinical care. Therefore, it is possible that this group of patients may have had a high pre-test probability of a bacterial infection. Restricting our study to this group of individuals also resulted in low penetrance of testing for viral illnesses, likely as a result of clinicians primarily suspecting bacterial infection during the course of clinical care. This limits the external generalizability of our results in other populations for whom bacterial infection may not be as strongly suspected.

Additionally, our study had low representation of adults under the age of 65 years. As noted above, age likely plays a role in the predictive capacity of point-of-care bacterial vs viral assays; our findings may have limited generalizability to younger adults based on the older age of our study population as a whole.

Finally, this study used pathogen-specific tests and blood cultures as a gold standard by which to compare MeMed BV assay results. However, in some cases, it is difficult to differentiate between a bacterial pathogen present as a commensal or colonizing species vs a pathogen causing an active, symptomatic infection. Our study reviewed results of multiple different blood bottles and assessed the likelihood of sample contamination or patient colonization based on this information; however, some study participants’ samples may have been misclassified. It is also worth noting that some pathogens, whether bacterial or viral, may not have been recovered using the standard methods used in the study and could thus represent false-negative results in our patient population.

### Conclusion

Several FDA-cleared point-of-care assays have been developed with the goal of helping providers in emergency departments and urgent care facilities differentiate between bacterial and viral infections, guiding them toward appropriate treatment. We investigated the use of the MeMed BV assay to aid clinicians in determining whether to order blood cultures. Our findings showed that the scores generated by the MeMed BV assay were more accurate in detecting viral infections than in identifying true bacteremia. However, the strong correlation between a high viral score and a negative blood culture can help physicians decide not to order a blood culture, thereby reducing the number of blood cultures ordered in the emergency room and, consequently, reducing antibiotic utilization.

The future of distinguishing bacterial from viral infections is shifting toward integrated diagnostic approaches. To combat antibiotic resistance, future diagnostics could utilize host immune response biomarkers, advanced molecular panels, and machine learning to provide rapid and accurate results at the point of care.

## SUPPLEMENTAL MATERIAL

Supplemental figure and tables jcm.00262-26-s0001.docx Fig. S1, and Tables S1 and S2.

ASM does not own the copyrights to Supplemental Material that may be linked to, or accessed through, an article. The authors have granted ASM a non-exclusive, world-wide license to publish the Supplemental Material files. Please contact the corresponding author directly for reuse.
