# Supplementary material for: Sensitivity and specificity of a rapid, point-of-care assay distinguishing bacterial from viral infections in acute care settings
Source: J Clin Microbiol. 2026 Oct 27;64(11):e00262-26. doi: 10.1128/jcm.00262-26 (PMC13753776; doi:10.1128/jcm.00262-26)
Supplement: Supplementary file 1 [file jcm.00262-26-s0001.docx]

**Supplemental Table 1**. Organisms recovered from samples of patients included in the study

| **Organisms Recovered** | **Among all samples** | **Samples with “high” bacterial activity on MeMedBV** | **Samples with “moderate” bacterial activity on MeMedBV** | **Source** |
| --- | --- | --- | --- | --- |
| *Aerococcus urinae* | 2 | 1 | 0 | Urine |
| *Bacteroides fragilis* | 4 | 4 | 0 | Blood/Tissue/Wound or abscess |
| *Bacteroides thetaiotaomicron* | 3 | 1 | 0 | Tissue/Wound or abscess/Fluid/Other (NOS) |
| *Candida albicans* | 5 | 3 | 1 | Sputum/Tissue/Wound or abscess/Fluid/Other (NOS) |
| *Candida dubliniensis* | 1 | 0 | 0 | Lavage |
| *Candida parapsilosis* | 2 | 1 | 1 | Tissue |
| *Citrobacter freundii* complex | 1 | 0 | 0 | Wound or abscess |
| *Citrobacter* species | 1 | 1 | 0 | Urine |
| *Clostridium difficile* | 1 | 1 | 0 | Fluid/Other (NOS) |
| *Corynebacterium aurimucosum* | 1 | 0 | 1 | Tissue/Other (NOS) |
| *Coryneform bacilli* | 11 | 7 | 1 | Tissue/Urine/Wound or abscess |
| *Cutibacterium acnes* | 6 | 4 | 1 | Blood/Synovial fluid/Tissue/Fluid |
| *Enterobacter cloacae* complex | 6 | 5 | 0 | Blood/Synovial fluid/Tissue/  Urine/Wound or abscess |
| *Enterococcus casseliflavus* | 1 | 0 | 1 | Blood |
| *Enterococcus faecalis* | 11 | 8 | 1 | Blood/Tissue/Urine/  Catheter/Fluid/  Other (NOS) |
| *Enterococcus faecium* | 5 | 4 | 1 | Tissue/Urine/Wound or abscess |
| *Escherichia coli* | 25 | 13 | 4 | Blood/Urine/Fluid |
| *Fusarium* species | 1 | 1 | 0 | Tissue/Fluid |
| *Klebsiella oxytoca* | 1 | 1 | 0 | Urine |
| *Klebsiella pneumoniae* | 8 | 5 | 1 | Blood/Urine |
| *Lancefieldella rimae* | 1 | 1 | 0 | Blood |
| *Lactobacillus* species | 3 | 2 | 0 | Tissue/Urine/Fluid |
| *Mycobacterium avium* | 1 | 0 | 1 | Sputum/Lavage |
| *Myroides* species | 1 | 1 | 0 | Blood |
| *Parvimonas micra* | 1 | 1 | 0 | Wound or abscess |
| *Pasturella multocida* | 1 | 0 | 1 | Tissue/Wound or abscess |
| *Prevotella denticola* | 2 | 2 | 0 | Bone/Cerebrospinal Fluid/Tissue/Wound or abscess |
| *Prevotella intermedia* | 1 | 1 | 0 | Wound or abscess |
| *Proteus mirabilis* | 5 | 4 | 0 | Blood/Bone/Tissue/Urine |
| *Pseudomonas aeruginosa* | 8 | 7 | 0 | Tissue/Urine/Wound or abscess/Fluid/Other (NOS) |
| *Serratia marcescens* | 1 | 0 | 0 | Synovial fluid/Tissue |
| Coagulase-negative staphylococci | 5 | 3 | 1 | Tissue |
| *Staphylococcus capitis* | 4 | 3 | 1 | Blood |
| *Staphylococcus aureus* | 21 | 13 | 3 | Blood/Bone/Synovial fluid/Tissue/Urine/Wound or Abscess/Fluid/Drainage |
| *Staphylococcus epidermidis* | 30 | 14 | 5 | Blood/Bone/Cerebrospinal fluid/Tissue/Tracheal aspirate/Urine/Other (NOS) |
| *Staphylococcus haemolyticus* | 2 | 2 | 0 | Tissue |
| *Staphylococcus hominis* | 7 | 4 | 2 | Blood/Urine |
| *Staphylococcus lugdunensis* | 1 | 0 | 0 | Urine |
| *Staphylococcus saprophyticus* | 1 | 0 | 1 | Urine |
| *Staphylococcus simulans* | 3 | 1 | 0 | Blood/Urine |
| *Staphylococcus warneri* | 1 | 1 | 0 | Blood |
| *Stenotrophomonas maltophilia* | 2 | 2 | 0 | Tissue |
| *Streptococcus agalactiae* | 4 | 1 | 3 | Blood/Sputum/Tracheal aspirate/Urine |
| *Streptococcus anginosus* group | 6 | 4 | 0 | Bone/Tissue/Wound or abscess/Fluid/Other (NOS) |
| Beta-hemolytic Streptococcus (Group G) | 1 | 0 | 0 | Blood |
| *Streptococcus intermedius* | 1 | 1 | 0 | Wound or abscess |
| *Streptococcus pneumoniae* | 1 | 1 | 0 | Blood |
| *Streptococcus pyogenes* | 1 | 0 | 1 | Wound or abscess |
| *Streptococcus viridans* group | 3 | 1 | 0 | Bone/Urine |
| *Trichophyton mentagrophytes* | 1 | 0 | 1 | Tissue |
| *Trichophyton rubrum* | 1 | 1 | 0 | Tissue |
| NOS: Not otherwise specified | | | | |

**Supplemental Figure 1.** Receiver-operator characteristic (ROC) curve, estimated by a maximum-likelihood probit model, showing estimated differences in area under the curve (AUC) according to patient age (panel A) and sex (panel B) at time of testing for bacterial (left) and viral (right) pathogens.

Panel A: ROC by age

Panel B: ROC by sex

**Supplemental Table 1A**

**Using ‘high likelihood’ categories only for the correct identification of bacteremia**

|  | High likelihood of bacterial infection | High likelihood of viral infection |
| --- | --- | --- |
| Blood culture testing performed | 99 | 14 |
| **Blood culture positive for bacterial infection** | **19** | **0** |
| Blood culture likely contaminated by commensal/non-pathogenic species | 10 | 1 |
| **Blood culture negative for bacterial infection** | **70** | **13** |
|  |  |  |

For **bacteremia** (excluding contaminated samples): true pos = 19, false pos = 70, true neg = 13, false neg = 0.

Sensitivity: $\frac{19}{19+0}$ = 100%. Specificity: $\frac{13}{13+70}$ = 15.663%.

**Supplemental Table 1B**

**Using ‘high likelihood’ categories only for the correct identification of viral infection**

|  | High likelihood of bacterial infection | High likelihood of viral infection |
| --- | --- | --- |
| Viral testing performed for a non-blood sample | 11 | 10 |
| **Sample positive for viral infection** | **2** | **10** |
| **Sample negative for viral infection** | **9** | **0** |

For **viral infection**: true pos = 10, false pos = 0, true neg = 9, false neg = 2.

Sensitivity: $\frac{10}{10+2}$ = 83.333%. Specificity: $\frac{9}{9+0}$ = 100%.

**Supplemental Table 1C**

**Using ‘high likelihood’ categories only for the correct identification of bacterial infection**

|  | High likelihood of bacterial infection  (n=99) | High likelihood of viral infection  (n=14) |
| --- | --- | --- |
| **Total cases positive for bacterial infection** | **61** | **4** |
| **Total cases negative for bacterial infection** | **38** | **10** |

For **bacterial infection**: true pos = 61, false pos = 38, true neg = 10, false neg = 4.

Sensitivity: $\frac{61}{61+4}$ = 93.846%. Specificity: $\frac{10}{10+38}$ = 20.833%.

**Supplemental Table 2A**

**Combining ‘high’ and ‘moderate’ likelihood categories together for the correct identification of bacteremia**

|  | High/moderate likelihood of bacterial infection | High/moderate likelihood of viral infection |
| --- | --- | --- |
| Blood culture testing performed | 131 | 36 |
| **Blood culture positive for bacterial infection** | **21** | **1** |
| Blood culture likely contaminated by commensal/non-pathogenic species | 13 | 3 |
| **Blood culture negative for bacterial infection** | **97** | **32** |

For **bacteremia** (excluding contaminated values): true pos = 21, false pos = 97, true neg = 32, false neg = 1.

Sensitivity: $\frac{21}{21+1}$ = 95.454%. Specificity: $\frac{32}{32+97}$ = 24.806%.

**Supplemental Table 2B**

**Combining ‘high’ and ‘moderate’ likelihood categories together for the correct identification of viral infection**

|  | High/moderate likelihood of bacterial infection | High/moderate likelihood of viral infection |
| --- | --- | --- |
| Viral testing performed for a non-blood sample | 16 | 15 |
| **Sample positive for viral infection** | **3** | **14** |
| **Sample negative for viral infection** | **13** | **1** |

For **viral infection**: true pos = 14, false pos = 1, true neg = 13, false neg = 3.

Sensitivity: $\frac{14}{14+3}$ = 82.35%. Specificity: $\frac{13}{13+1}$ = 92.857%.

**Supplemental Table 2C**

**Combining ‘high’ and ‘moderate’ likelihood categories together for the correct identification of bacterial infection**

|  | High/moderate likelihood of bacterial infection  (n=131) | High/moderate likelihood of viral infection  (n=36) |
| --- | --- | --- |
| **Total cases positive for bacterial infection** | **79** | **12** |
| **Total cases negative for bacterial infection** | **52** | **24** |

For **bacterial infection**: true pos = 79, false pos = 52, true neg = 24, false neg = 12.

Sensitivity: $\frac{79}{79+12}$ = 86.813%. Specificity: $\frac{24}{24+52}$ = 31.578%.
